# Relevance of K13 mutations for malaria control and elimination program

**DOI:** 10.1186/1475-2875-13-S1-O40

**Published:** 2014-09-22

**Authors:** Frédéric Ariey

**Affiliations:** 1 Institut Pasteur, Paris, France

## 

The K13 gene mutations have been found to be associated with artemisin resistance in Cambodia, although the relevance of these mutations on artemisinin resistance in other endemic areas have to be better documented. We present here new data on K13 mutations and on the artemisinin resistance outside Cambodia. We propose to open the discussion about how these new findings have to be interpreted regarding the emergence and spread of artemisinin resistance and their usefulness to identify and prevent the spread of resistant strains in order to reach the malaria control and elimination global program goal.

